# Community participation: Lessons and challenges of the 30 years of health councils in Brazil

**DOI:** 10.7189/jogh.11.03061

**Published:** 2021-03-27

**Authors:** José Patrício Bispo, Mauro Serapioni

**Affiliations:** 1 Multidisciplinary Health Institute, Federal University of Bahia (UFBA), Vitória da Conquista, Brazil; 2 Center for Social Studies, University of Coimbra (UC) and visiting professor Federal University of Santa Catarina (UFSC). Coimbra, Portugal

In 2020, Brazil celebrates 30 years of the national health system creation and institutionalization of community participation in the health care sector. The Unified Health System (*Sistema Único de Saúde* – SUS) is a universal access health system financed by general taxes, and one that has consistently progressed towards enabling comprehensive health care for the Brazilian population [1]. However, the SUS faces consistent difficulties, especially insufficient public funding [2], as well as strengthening of the private sector, and persistent social inequalities in the country [3].

The origin of the SUS date back to the Brazilian Sanitary Reform (BSR) movement, a broad social movement in the 1980s that defended Brazilian re-democratization, defined health as a social right, and led to the creation of the national health system. As other Latin American countries were moving towards segmented health care models, the BRS movement fought to transform the fragmented and exclusive system and to assure health as a citizenship right [3]. After a decade of struggle, the Federal Constitution of 1988 established health as a universal right and the State was given the responsibility of ensuring health care. Thus, the genesis of SUS was strongly influenced by the strength of the social mobilization experienced in the country.

In 1990, Law 8142 instituted the creation of health councils (HC) as part of the organizational structure of the health system. The social bases that influenced the institutional design of SUS advocated for involving the general population in the decision-making process and in controlling health policies. Another element influencing the creation of HC was the expectation of maintaining a state of social mobilization. Thus, the HC would promote citizen education and act as a basis for supporting democratic principles in health. In this article, we discuss the lessons and challenges of institutionalizing community participation in the 30 years of the SUS.

## LESSONS FROM COMMUNITY PARTICIPATION IN THE SUS

Important lessons were learned from the Brazilian experience with community participation in the health sector. Establishing community participation as a principle and institutionalizing HC as part of the SUS structure represents the political decision of Brazilian society to not only have momentary social participation, but also make it a permanent and necessary element that can contribute to societal development. We consider capillarity, inclusiveness, and political influence capacity as important lessons learned from the Brazilian experience in health participation.

Capillarity refers to the expansion of participatory bodies across Brazil. The association between the institutionalization of community participation and the decentralized organization of the SUS improved the expansion of participatory bodies in all federated entities at the three government levels, federal government, states, and municipalities. There are 5570 municipal HC in Brazil, 26 state HC, 36 district indigenous HC, and one national HC [4]. Additionally, many municipalities created local HC in primary care centers and hospitals, exhibiting extensive capillarity of community participation in Brazil from local to national levels.

Inclusiveness corresponds to the involvement of multiple stakeholders in the participatory process. HC are organizations that have collective representations with the following composition: 50% community representation institutions, 25% health professionals, 12.5% managers, and 12.5% service providers. This architecture seeks to represent several interests directly related to health care provision [5], that bring together community, besides technical-specialized, scientific, and governance components. Even by adding several stakeholders, it maintains the primacy of its community character, with social representatives occupying half of the HC seats. In this sense, the experience of SUS participation can be considered strongly inclusive and one that greatly values social participation.

Political influence capacity refers to recognizing HC as a decision-making space in health. As defined by Law 8142, HC is responsible for deliberating on health policies. This law also establishes that HC decisions must be approved by the head of the executive branch. This represents an important mechanism to share power between the State and society, as decision-making is no longer the exclusive responsibility of health managers. Deliberation in the Brazilian health system includes making HC as an appropriate public sphere, thereby allowing different social segments to debate and negotiate on health policies that are better suited for the population.

In this sense, HCs stand out not only for its capillarity and inclusiveness but also for being a potential space to control health actions and budget execution in the sector. HCs are characterized as bodies that aid community empowerment [6], that stand out from the nonpermanent participatory experiences of other countries, which provide limited inclusiveness and have no decision-making power, only consultative nature.

## COMMUNITY PARTICIPATION CHALLENGES IN BRAZIL

Despite the progress achieved, HC presents important challenges to become an effective participatory space. We highlight the four orders of performance problems.

**Figure:**
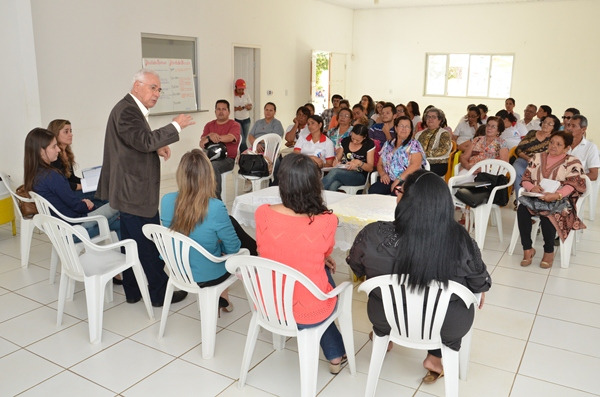
Photo: Health Council of Primary Health Center in Vitória da Conquista, Bahia Brazil (from the official website of the City of Vitória da Conquista, used with permission). Available at: http://www.pmvc.ba.gov.br/wp-content/uploads/SEC_2058.jpg.

First, the challenges related to community mobilization and involvement. It has been difficult to maintain high levels of community engagement over the past three decades. The institutionalization of participation was not enough to keep the civil society mobilized to defend the health system. Many HC find it difficult to function regularly, mainly due to the weakening of civil society participation. Because of a decrease in community involvement, managers and service providers play a leading role in conducting the activities, controlling the discussion agenda, and have a strong influence on the attitude of other representatives.

Second, the challenges related to the bureaucratization and technification of participation. HCs have a wide range of attributions concerning the deliberation on health policies, monitoring, and inspecting the implementation of these health policies, analyzing accounting balance sheets, and promoting social mobilization [5]. The representatives’ performance has a strong bureaucratic nature and presupposes specialized accounting and technical knowledge. Excessive participatory technification has weakened the community component, moving social movements away.

The third challenge is related to effectiveness. Participation requires the ability to influence the health agenda [7,8]. Although HCs have legal responsibility and possess essential decision-making characteristics, they do not have power to influence the managers’ decisions in all situations. Often, decisions are made in offices and taken to the HC only for formal approval. Thus, these entities end up with a figurative decision-making role. Effective decision-making power constitutes a major community participation challenge in Brazil.

Fourth, the challenges arising from the weakening of the SUS and current ultraliberal policies. Over the past 30 years, SUS has never had enough funding, presenting structural problems that hinder access and affect health care quality [1,2]. Since 2016, the situation has been further aggravated by the strict fiscal austerity programs implemented in Brazil. Silva et al^2^ affirm that the Bolsonaro government has imposed successive direct attacks on SUS that threaten the universal character of the Brazilian health system. A weakening of the health care system causes a decrease in community participation as social segments start to discredit the capacity of the public health system.

## CONCLUSIONS

The Brazilian HC is a mechanism of democratic innovation that can be considered a successful arrangement for community participation in health systems. Capillarity, inclusiveness, and deliberative attribution enabled the establishment of a participatory structure distributed throughout the country, which included community representatives in the decision-making process. After 30 years, the SUS experiences a process of social demobilization that threatens the effectiveness of HC and the SUS itself. Community participation is the only way to strengthen participatory bodies. We highlight the need to resume the process of social mobilization and the democratic project in health.
